# Implementing and Sustaining Brief Addiction Medicine Interventions with the Support of a Quality Improvement Blended-eLearning Course: Learner Experiences and Meaningful Outcomes in Kenya

**DOI:** 10.1007/s11469-022-00781-6

**Published:** 2022-05-23

**Authors:** Veronic Clair, Kaitlin Atkinson, Abednego Musau, Victoria Mutiso, Edna Bosire, Isaiah Gitonga, Will Small, David Ndetei, Erica Frank

**Affiliations:** 1 Africa Mental Health Research and Training Foundation, PO Box, Nairobi, 48423-00100 Kenya; 2 Faculty of Health Sciences, Centre for Applied Research in Mental Health and Addiction (CARMHA), Simon Fraser University, 8888 University Drive, Burnaby, BC V5A 1S6 Canada; 3 School of Population and Public Health, The University of British Columbia, 2206 East Mall, Vancouver, BC V6T 1Z3 Canada; 4 NextGenU.org, PO Box 7, Clear Lake, WA 98235 USA

**Keywords:** Health services administration and management, Medical education and training, Mental health, Substance misuse, Brief intervention, Primary care, Lay health worker, Quality improvement, Qualitative research, eLearning, Blended courses, Addiction

## Abstract

Quality improvement methods could assist in achieving needed health systems improvements to address mental health and substance use, especially in low-middle-income countries (LMICs). Online learning is a promising avenue to deliver quality improvement training. This Computer-based Drug and Alcohol Training Assessment in Kenya (eDATA-K) study assessed users’ experience and outcome of a blended-eLearning quality improvement course and collaborative learning sessions. A theory of change, developed with decision-makers, identified relevant indicators of success. Data, analyzed using descriptive statistics and thematic analysis, were collected through extensive field observations, the eLearning platform, focus group discussions, and key informant interviews. The results showed that 22 community health workers and clinicians in five facilities developed competencies enabling them to form quality improvement teams and sustain the new substance-use services for the 8 months of the study, resulting in 4591 people screened, of which 575 received a brief intervention. Factors promoting course completion included personal motivation, prior positive experience with NextGenU.org’s courses, and a certificate. Significant challenges included workload and network issues. The findings support the effectiveness of the blended-eLearning model to assist health workers in sustaining new services, in a supportive environment, even in a LMIC peri-urban and rural settings.

The Global Burden of Diseases, Injuries and Risk Factors Study 2017 estimates that globally, more than 175 million people suffer from alcohol or other substance-use disorders (excluding tobacco)(James et al., 2018), while 165 million people use tobacco (Reitsma et al., 2017). The use of tobacco and other psychoactive substances causes immense health, social and economic consequences in high income countries (HICs) as well as low- and middle-income countries (LMICs) (Lund et al., 2011; Marangu et al., 2014; Patel et al., 2015; World Health Organization, 2018). In LMICs, mental health disorders and substance-use-related morbidity and mortality result in a higher burden of disease than HIV/AIDS, tuberculosis, diabetes, and transport-related injuries combined, but receive far less attention and funding (Lim et al., 2013) with considerable deficiencies in services availability (Lora et al., 2020). A lack of trained providers further challenges program implementation to address these conditions, along with stigma, weak governance, and difficulties in shifting practice patterns (Marangu et al., 2014; Patel et al., 2013, 2015; Saraceno et al., 2007). Although more research is needed on how to best address mental health and substance use, especially in LMICs, quality improvement (QI) methods could assist in achieving needed health systems improvements (Kruk et al., 2018). There is emerging evidence that participation in QI training and collaborative groups are promising approaches (Garcia-Elorrio et al., 2019; Rowe et al., 2018). For example, ‘Project Fives Alive!’ in Ghana used QI methods to improve child health services countrywide, causing under-five mortality rates to decline significantly (Singh et al., 2016).

Blended-eLearning (online learning complemented with face-to-face activities) could offer an effective and potentially cost-effective option to provide relevant training (George et al., 2014; Hew & Cheung, 2014; Lewis et al., 2014; Liu et al., 2016; Maloney et al., 2012; Maloney et al., 2015; Marrinan et al., 2015; Sandars, 2010; Shorbaji et al., 2015; Sinclair et al., 2015; Walsh et al., 2010), even in LMICs, as technological access and user-capability improve (Bahia & Suardi, 2019; Dagys et al., 2015; Gomez, 2014; Kebaetse et al., 2014; Marrinan et al., 2015; Parent & Cruickshank, 2009; Sissine et al., 2014; The World Bank, 2020). To date, most eLearning research relates to imparting clinical competencies, with few studies on teaching QI or leadership, and most of those limited studies focus on the Institute for Health Care Improvement (IHI) online Open School (Bonnes et al., 2017; Mehta & Sharma, 2018; Suliman et al., 2018). Therefore, much remains unknown about the effectiveness of eLearning in this field (Tudor Car et al., 2018), especially in LMICs. A gap this study hopes to address as part of the Computer-based Drug and Alcohol Training Assessment in Kenya (eDATA-K), a multi-phase mixed-methods research program.

eDATA-K uses developmental evaluation methods to optimize and assess the impact of an innovative approach to building human capacity to address the health services gap related to substance use, using the NextGenU.org model (Frank et al., 2016; Galway et al., 2014; Madhok et al., 2018; Rossa-Roccor et al., 2017). NextGenU.org creates free, online, competency-based courses with screened open educational resources complemented with contextualization, peer- and mentored-activities, quizzes and final exam evaluation, and certificates to support trainees’ acquisition of key competencies in a given field. eDATA-K showed that NextGenU.org courses imparted trainees with the necessary competencies to screen and address substance use in primary care. The courses also contributed to decreasing healthworker’s stigma toward those with substance use disorders (SUD) (Clair et al., 2016a, b, c, 2019, 2022a). eDATA-K demonstrated that community health workers delivering the Alcohol, Smoking and Substance Involvement Screening Test (ASSIST) (Humeniuk et al., 2012, 2008, 2010) were effective in supporting patients to decrease their alcohol consumption as much as clinicians delivering a full ASSIST linked brief intervention (Clair et al., 2016b, c, 2022b). Furthermore, in this LMIC context, patients receiving either intervention decreased their alcohol use more than those receiving brief intervention in HICs (Clair et al., 2016b; Humeniuk et al., 2018; Kaner et al., 2018).

The last phase of eDATA-K and the object of this article studied the use and impact of the Practice Change and Quality Improvement (PCQI) course. The PCQI course was conceived to support health workers in sustaining the new services in regular clinical practice. The PCQI study research questions are: 1) what is the experience of community health workers, support staff, health officials, and clinicians taking the PCQI course? 2) how has it impacted participants’ knowledge, attitudes, and skills? And 3) what is the course’s impact on integrating and sustaining the new services into regular clinical practice?

## Methods

From 2012 to 2016, eDATA-K used a developmental evaluation approach to dynamically assess and adjust the NextGenU.org blended-eLearning courses related to substance use, including the PCQI. Developmental evaluation approaches are most useful for unstable contexts, complex problems, and insufficient consensus on the best programs or policies (Patton, 2011). Unstable contexts, complex problems, and insufficient consensus of programs or policies are issues plaguing the use of blended-eLearning in LMICs (Kebaetse et al., 2014; Liu et al., 2016; Maloney et al., 2012), the management of SUDs globally (Bartlett et al., 2014), and the strengthening of health systems (Armstrong et al., 2017; Boonyasai et al., 2007; Garcia-Elorrio et al., 2019; Rowe et al., 2018; Tudor Car et al., 2018).

**Fig. 1:**
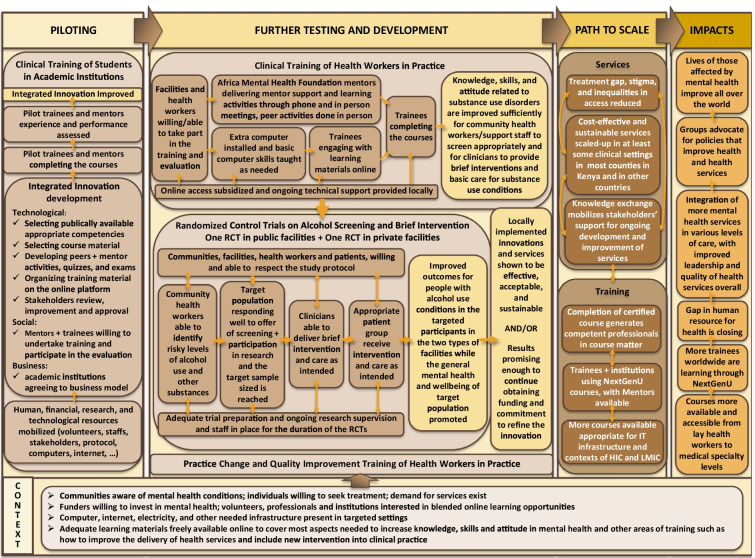
eDATA-K theory of change modified post-randomized control trial

**Fig. 2:**
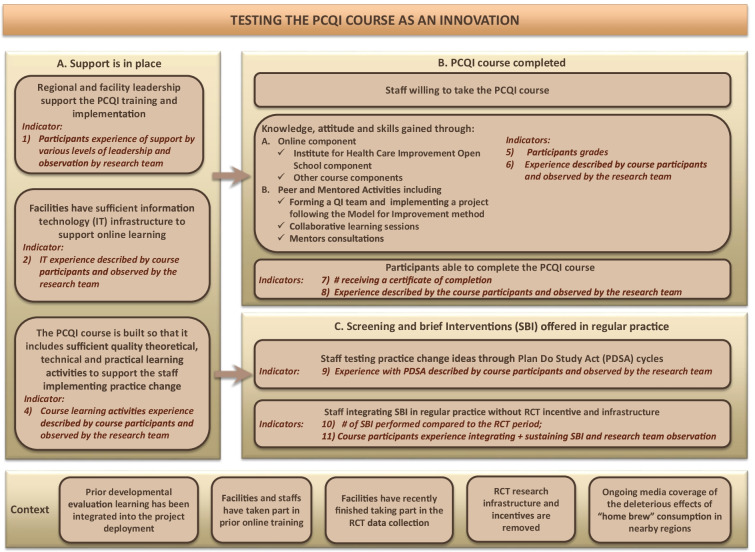
Theory of change for eDATA-K Practice Change and Quality Improvement (PCQI) course’s study

Consistent with evaluation methodologies, a theory of change (De Silva et al., 2014) was created through workshops in 2012–2013 in collaboration with Kenyan national, regional, and local decision-makers and researchers. A theory of change is an implementation model where end-users and decision-makers are involved in hypothesizing what factors are needed for the desired change to happen, and what indicators and data are needed to verify the assumptions and ascertain the impact of the change. It is not the elaboration of a scientific theory. The eDATA-K theory of change components, assumptions, and indicators were heavily influenced by the one developed by the funder of the research program, Grand Challenges Canada, and by published literature on practice change, implementation science, and substance use (Damschroder & Hagedorn, 2011; Garner, 2009; Massoud et al., 2012; Miller et al., 2006; Peters et al., 2013). The theory of change was further adjusted following the completion of the prior phases of eDATA-K (Fig. 1) and further detailed to guide and support the research process for the PCQI study (Fig. 2). The theory of change postulates that with appropriate support, in the current Kenyan context, participants would be willing to take and able to complete the PCQI course, leading to mastery in the course’s competencies. Their newly acquired knowledge, skills, and attitudes regarding leadership and quality improvement should allow them to integrate and sustain the new services in practice even after removing the randomized control trial incentive and research infrastructure of the last eDATA-K phase.

The PCQI study ran from January to September 2015. It used a descriptive thematic methodology (Sandelowski, 2000) to provide a rich phenomenological interpretation of health care workers’ experiences with the course and its impact on their knowledge, skills, attitudes, and practices. Data were collected through field observation, participant’s course information from the eLearning platform, focus group discussions (FGD), and key informant interviews (KIIs), linking the observations back to the theory of change (Onwuegbuzie et al., 2009; Snape & Spencer, 2003).

### The PCQI Course

The course was designed to be a blended-eLearning competency-based course in QI and leadership for the improvement of mental health and substance use services (Afsaneh, 2014; Armstrong et al., 2017; Baartman & de Bruijn, 2011; Blood-Siegfried, 2008; De Leeuw et al., 2016; George et al., 2014; Means et al., 2009; Starr et al., 2016; Wong et al., 2010). Per NextGenU.org’s methodology (Rossa-Roccor et al., 2017), qualified resources (only from universities, professional societies, governments, or peer-reviewed journals) were curated and assembled to teach competencies identified as necessary to effect change in clinical practice, including leadership and QI skills. PCQI competencies were derived from the Canadian College of Health Leaders (Canadian College of Health Leaders, and LEADS Collaborative, 2010), the UK National Health System Leadership Academy (NHS Leadership Academy, 2012), and IHI (IHI, 2003, 2015) (Table 1). The PCQI course includes online didactic reading and quizzes for each module, and predominantly in-person peer and mentor activities, serving to impart and assess participants’ competencies (Harrison & Mitchell, 2006). The online component includes four modules with learning materials from high-quality sources, such as the IHI Open School’s Improvement Capability courses (IHI Open School, 2012, 2020). A proportion of the learning material was available offline. The course was designed to maximize this. But, due to the quality of the IHI content, it was felt that it was better to use this resource even if it was less easy to access. Peer and mentored activities consisted of (1) establishing a quality improvement team with regular local facility quality improvement meetings using Plan, Do, Study, Act cycles; (2) four collaborative learning sessions joining representatives from participating facilities, where mentors reviewed course content and offered a forum to share progress, challenges, and lessons learned; and (3) monthly mentor visits with phone calls and emails in between visits, as needed, to support trainees. The model for the PCQI relied on eDATA-K researchers acting as mentors. Trainee assessment, online course evaluation, and self-evaluation were based on various evidence-based frameworks (Baartman & de Bruijn, 2011; Blood-Siegfried, 2008; Halawi et al., 2009; Harrison & Mitchell, 2006; Means et al., 2009).

**Table 1:** Adjusted competencies, from IHI, the NHS Leadership Institute, and the Canadian College of Health Leaders, covered by the Practice Change and Quality Improvement (PCQI) course

Module	Competencies	Learning objectives
1. Understanding Leadership and Quality Improvement Contribution to Health Care	• Understand leadership and quality improvement’s contribution to health care	• Understand the need for leadership in medicine, including to improve clinical care • Understand the need and develop skills for self-assessment of leadership • Develop a clear understanding of the model of improvement in healthcare
2. Managing Services and Planning Change Initiatives	• Demonstrate effectiveness in planning the delivery of health care initiatives • Demonstrate effectiveness in managing resources • Demonstrate effectiveness in managing people • Demonstrate effectiveness in managing performance	• Understand the concept of institutional leadership and the place of planning and policy in healthcare delivery • Understand the role of management (people and resources) in the delivery of high-quality services in mental health • Understand the role of working with all stakeholders and the role of networking for the organization in delivering healthcare
3. Improving Services	• Demonstrate effectiveness in implementing quality improvement, especially as it relates to mental health and substance use disorders services	• Identify areas in the healthcare system that require changes through several methods, e.g., critical evaluation • Develop innovations/change ideas that can lead to improvement • Facilitate the transformation of health services while observing patient safety • Use the IHI model for improvement (using QI methodologies in developing and implementing changes through PDSA cycles) • Have some skills to measure the success of implementing changes in the health sector
4. Working with Others	• Demonstrate effectiveness in developing networks and building and maintaining relationships • Demonstrate effectiveness in encouraging contribution and motivating people • Demonstrate effectiveness in working within teams	• Have a clear understanding of how to build and maintain a good working relationship with all team members • Encourage the participation of both providers, beneficiaries, and other stakeholders in decision making in the healthcare delivery process • Demonstrate the capacity to work with multiple stakeholders as a team • Develop an understanding of ways on how to deal with resistance to changes in the healthcare delivery process • Develop practical solutions that can address most of the challenges that may arise in the process of implementing change in the healthcare delivery process

### Participants

eDATA-K was implemented in public facilities in two counties selected for their geographic accessibility, willingness to engage in the research, comparability, and population characteristics. Each county included about 1 million Kenyans living in relatively typical suburban and rural conditions. This study focused on the county that received the course first, immediately after completing the randomized control trial phase; the other county, which received a delayed implementation, is the object of a separate study.

The QI course was offered to all staff members of the five health facilities, which had completed prior phases of eDATA-K, and health officials in relevant leadership roles. The participating facilities were three rural health centers and two outpatient hospital departments, which serve as primary care clinics in their towns. Facilities were provided with one computer and pre-paid online codes to access the internet. Participation in the PCQI  course and the accompanying study was voluntary. Participants did not receive remuneration to take the course. However, participants most involved in their local QI teams were prioritized to represent their team at collaborative learning sessions and were reimbursed for attendance-related transportation and meals.

### Data Collection

Per developmental evaluation practices, the research team was integral in the innovation’s refinement and optimized implementation. The research team conducted site observations before, during (at least monthly in person, plus frequent phone calls with participants in between to check-in and problem-solve around course progression, successes, and challenges), and after the course. The research team also conducted several regional stakeholders’ meetings in preparation for, during, and at the end of the QI phase of eDATA-K. Since mentors were included in the research team and present at all learning sessions, mentoring and field observation activities frequently co-occurred. The research team also collected documentary evidence of the new course’s impact on services implementation from the county’s planning documents. Participation in these activities generated valuable insight into course participants’ experience and the course’s impact on knowledge, attitudes, skills, and the services’ sustainability. Insights gained through these observations and interactions were recorded in staff field reports and meeting minutes. The NextGenU.org training platform collected registration and other information such as participants’ login, grades, course evaluation and self-evaluation forms, and course completion status.

Participants for FGDs were selected through purposive sampling to obtain representation from different genders, facility types, and professional backgrounds (clinicians, health officials, community health workers, and support staff). All invited subjects agreed to participate, but two individuals were unavailable on the date of one FGD. They were offered to take part in key informant interviews (KIIs) at a later date. Participants in FGDs and KIIs received a small financial compensation for their time.

FGDs and KIIs followed a semi-structured interview guide, reviewed by local experts for cultural appropriateness. The FGDs and KIIs were conducted in-person in a mixture of English and Kiswahili (to facilitate participants’ expression), were audio-recorded, and were moderated by qualitative researchers.

### Ethics Statement

The University of British Columbia and the Kenya Medical Research Institute ethics boards approved the research project. Course participants provided informed consent. Participants in FGD and KII provided separate and specific informed consent, including for audio-recording, transcription, and translation.

### Data Analysis

The co-Principal Investigator, PhD-trained in mixed methods studies, obtained and analyzed the learning platform data using descriptive statistics. Two master-level qualitative researchers (one Canadian and one Kenyan), not involved in course delivery, conducted a thematic analysis of FGDs and KIIs transcripts using a constant-comparison method with NVivo (Doody et al., 2013; Snape & Spencer, 2003). They independently immersed themselves in the data to inductively code information into research-relevant themes/sub-themes, repeating until saturation. After comparing codes, the qualitative researchers arrived at a final coding framework with the co-Principal Investigator. Data were revisited by each researcher for final independent coding, with discrepancies resolved through discussion. Final coding frameworks and interpretation were refined by the entire eDATA-K research team, adding insight from those most involved in field observations and allowing professionally diverse researchers (public health, medicine, psychology, social sciences, and health administration) to enrich interpretations. Researchers discussed findings with course participants and leaders as part of the course graduation ceremony. Such dissemination and co-construction of knowledge further refined and validated data interpretation.

## Results

Twenty-four front-line practitioners (13 nurses or clinical officers and 11 community health workers or support staff; nine males and fifteen females) in two hospitals and three community clinics engaged meaningfully with the course and attempted the final exam. Twenty-two graduated (average grade: 82%). One community health worker and one clinician failed with grades < 60%. All twenty-two demonstrated mastery in the course competency based on performance in mentored-activities. Sixteen health workers logged into the course but dropped out due to workload, staff transfer, and difficulties accessing the internet. Respectively ten and seven participants completed the voluntary eLearning platform course evaluations and self-evaluation, with answers to multiple-choice questions reported in Table 2 and open-ended responses included in the qualitative analysis.

**Table 2:** Course evaluation and self-evaluation results from NextGenU.org online course platform

	% agreement
**Course evaluation (*n*** **= 10)**	
**Competencies and learning objectives**	
1. The course competencies were clear	100
2. There were enough learning materials (e.g., recorded audio/video lecture, PDF, etc.) to study each competency in appropriate depth	80
3. The learning objectives (the goals) for the learning materials were clearly defined	100
** Learning materials**	
4. The content of the learning materials was at the right level of difficulty	90
5. The learning materials contributed to my learning	90
6. The learning materials were of high quality	90
7. The learning materials were clearly connected to the learning objectives	100
**Mentored-activities**	
8. I enjoyed working on mentored-activities	100
9. The instructions for mentored-activities were clear	50
10. The mentored-activities contributed to my learning	60
**Multiple choice quizzes and exams**	
11. The quizzes linked to each learning objective contributed to my learning	100
12. The multiple-choice questions for the quizzes and final exam were understandable	90
13. There was enough time to complete the final multiple-choice exam	100
**Peer to peer activities**	
14. I enjoyed working on peer-activities	100
15. The instructions for peer-activities were clear	100
16. Peer-activities contributed to my learning	100
**Technical aspects**	
17. I used the Google Translator tool	80
18. I had sufficient access to the appropriate hardware and software to complete this course	80
19. If I had to email a question to NextGenU.org staff, I received a timely response	60
20. It was easy to access learning resources online	100
21. It was easy to interact with peers online	80
22. It was easy to upload work to the NextGenU.org course website	100
23. NextGenU.org clearly outlined what kinds of technical resources were necessary to complete this course	100
24. My questions about the course and/or the website were answered on NextGenU.org’s informational pages	70
25. The NextGenU.org website was easy to navigate	90
**Overall**	
26. In the future, I would prefer to take another NextGenU.org course than a traditional (classroom) course	90
27. I would recommend this course to a peer	100
28. I would take another NextGenU.org course in the future	100
29. I believe the education I received from NextGenU.org is of equal or better quality as the education from a traditional university in my home region	100
**Course self-evaluation** (*n* = 7)	
1. I feel confident that I gained substantial knowledge about the subject matter	100
2. I spent an adequate amount of time reviewing the online learning resources in order to learn the material	100
3. I gained the knowledge and skills necessary to acquire competency in the subject matter	100
4. I now understand how professionals in this discipline think about issues	100
5. I felt comfortable learning independently online	100
6. I felt comfortable with the amount of effort I put into the course	100
7. I felt comfortable interacting with my peers	100
8. I enjoyed the time I spent on this course	100
9. I completed all of the tasks my mentor assigned to me	57
10. I learned new skills that will be useful to me in the future	100
11. I participated in some or all of the optional activities	43
12. I was willing to take on additional responsibilities from my mentor as appropriate	71
13. I actively participated in online discussions	57
14. I communicated effectively with my mentor	29
15. I communicated effectively with my peers	100
16. I put in my best effort to complete the learning activities	100
17. I listened to and incorporated the feedback I received from others	100
18. I was able to solve the problems that I encountered throughout the course	100

Eighteen trainees participated in the FGDs or KIIs. Both FGD had 8 participants (6 females and 2 males), with representation from all participating facilities; one was conducted with community health workers and one with clinicians. Two nurses in leadership positions (one female and one male) could not attend that FGD and participated in KIIs instead.

### Impact of Participation in the Course on Integrating and Sustaining the New Services

As part of PCQI learning activities, all five health facilities established a QI team that worked towards improving and sustaining the new substance-use services. All teams functioned for the duration of the 8-month follow-up period and intended to continue working on QI after that. All teams successfully sustained the provision of screening and brief intervention in their facilities. PCQI participants tracked the services they offered as part of their QI work and reported screening 4591 people, with 575 qualifying for and receiving a brief intervention, with complications and comorbidities addressed as needed. Data from field observations, FGDs, and KIIs confirmed that teams achieved this by using their newly acquired competencies in leadership and QI methods. New services were integrated into the regular workflow without extra human or financial resources. Participants described that screening and offering brief intervention was now “part of their daily routine.” They stated: “We have become addicted [to screening and brief intervention] (all laugh).” to the extent that if they did not offer these services, “they felt they were missing something.”

Challenges with sustaining services were similar to those associated with completing the course, including infrastructure, workload, and staff transfer issues. Space was an issue when community health workers and support staff wanted to continue delivering screening services, but there was no official clinical room dedicated to that work. Some eDATA-K facilities had pre-existing staff shortages or long wait times, or both. Such facilities’ participants reported spending extra time with patients who used tobacco, alcohol, or other substances, which caused the wait time to increase sometimes. Clinicians reported working overtime, without pay, because of their devotion to delivering these services. Staff members reported collaboratively developing and testing improvement and change ideas to mitigate the increased workload, such as shortening the screening tool by targeting fewer substances. They proceeded iteratively until a change idea was successful in enabling the team’s goal.

The change ideas [are] very easy to adapt in the communities and those that are hard to adapt to, we can go back and change them. Also, the idea of the fishbone where you question something until you get an answer or if you cannot get an answer, you change it. That has really helped. I am thus able to apply these things in my day-to-day work as I carry on with my routine work in mental health. [Makueni Clinician]

Using new PCQI skills, one of the transferred front-line staff generated enough support to continue offering screening and brief intervention services in his new community health center, even though no one else had been involved in addressing substance use before. He did so without receiving an official mandate. Another staff member was promoted to a leadership position and asked to use his new clinical competencies and leadership skills to implement substance-use-related services in his new work setting. And for teams who received new, untrained staff members, the rest of the local QI team trained and mentored them in substance-use-related knowledge, skills, and attitudes.

Participants explained that the course enabled them to apply their new competencies from the PCQI course to work on issues beyond SUDs: they felt it was important to expand and scale up positive changes, and they had the confidence to do so. Some participants also shared their new knowledge and skills with other community members and with family members to effect even broader change.

Most significantly, the county leadership team found the project to be so successful that they included budgeting for a specialized SUD program in their planning documents, in part to address the issue of transfers. And under the leadership of one head nurse participant, the county also created a referral clinic at the hospital outpatient department for individuals requiring additional interventions to manage their SUD, mental health complications, or comorbidities.

### Participants’ Experience with the PCQI Course

Theory of change Component A postulates that participants need adequate leadership support, IT infrastructure, and course quality to complete the course and use their acquired competencies to achieve a sustainably improved service. Participants felt supported by leadership, mainly through leaders’ attendance of the course graduation ceremony, where leaders of all levels expressed (1) project alignment with the county’s vision of improving mental health services, (2) congratulations to graduates, and (3) encouragement to continue with the next phase on QI. This was particularly obvious during the graduation ceremony, as per field observation. Most participants expressed that providing a computer in each facility and free internet access was instrumental for course engagement and completion (FGD and KII, confirmed by field observation). Course evaluation, self-evaluation, and participants’ feedback in the FGD and KII support the assumption: offering a high-quality course, the third critical theory of change-supportive element, is key. In the eLearning platform course evaluation, the mentored-activities were reported as contributing to the participants’ learning in a lower proportion than other course components. There seemed to have been confusion in the eLearning platform course evaluation survey as to what constituted the mentored-activities covered by that survey, with participants commenting on the mentor support and initial mentored-activities described in the online platform. But communication with mentors through the eLearning platform was put aside in favor of e-mails, phone calls, and in-person sessions (an adjustment to the course established as part of the developmental evaluation practices). While some participants wished mentors were more responsive, others expressed they felt well supported to complete the courses, even by mentors. Participants also stated they found the regular mentorship visits and the in-person learning sessions extremely useful. Field observations strongly support that the collaborative learning sessions constituted an essential part of the mentored-activities, with high-quality and highly valued mentor’s support provided during the sessions. Trainees reported it was an essential element to acquire competencies and was a key enabler of their success in sustaining the services. Trainees valued those sessions so much that they asked for an extra learning session than the initially three planned for the course, and the mentors obliged them.

Theory of change’s Component B (completing the QI course and gaining competencies through new knowledge, attitudes, and skills) postulates that this is possible even without dedicated time away from their duties or financial compensation when the elements of Component A are in place. Our findings confirmed that aspects of Component A contributed to participants’ willingness to engage with the course, with the two strongest motivators being (1) previous positive learning experiences with other eDATA-K/NextGenU.org courses and (2) a desire to acquire knowledge and skills for sustaining new services in their clinical practices. Participants expressed feeling empowered to address substance use upon completing the eDATA-K/NextGenU.org clinical courses. Because taking the clinical courses translated into positive clinical experiences (e.g. successfully helping their patients and their friends/family members to decrease or abstain from using substances and becoming healthier), it encouraged staff members to register for and complete the PCQI course and to put the learning in practice to sustain the services they valued.

Course completion certificates provided by IHI/NextGenU.org and recognition at the graduation ceremony were mentioned as other important motivators to enroll in and complete the PCQI course. The interactive features of the PCQI course, such as peer learning and mentorship, QI group work, and the online content (especially the detailed examples and videos), were mentioned as other motivating and helpful components. Participants appreciated the opportunity to test/validate their understanding of the course content, practice skills, and learn through different teaching modes. Participants considered the PCQI language easier to understand than the clinical courses’ text, especially for less-formally trained participants such as community health workers and support staff.

The practice support [PCQI course] is very interesting, it has very nice examples and the language it is put in is quite encouraging, and it, therefore, builds you up to be able to face people with drug and substance abuse in a better way with very smart examples. [Makueni Clinician]

Course participants reported they were delighted with the knowledge and skills acquired, giving them a boost in self-confidence and work performance in general. They linked their learning to their success with sustaining the services. Participants found the modules that focused on leadership, managing errors and mistakes, and time management was most helpful in building confidence in managing change.

However, course completion was not without challenges, particularly limited infrastructure, staff workload, and staff transfers. In terms of infrastructure, despite the provision of a computer and staff members frequently completing modules together, occasionally, two (or more) individuals wanted to complete different modules at the same time.

So then you find that the sharing of computers is a challenge because it is one and the time I break from my normal duties, maybe during lunchtime and that is the time I want to peep in the computer and learn something on roles, maybe tackle my exams. You find it is a challenge because, at the same time, he [someone else] got a chance to come and read. [Screener]

Also, the IHI component (not developed for low Internet bandwidth usage, unlike other course components) was challenging to complete in one low-bandwidth facility. Furthermore, the computer was typically located in clinical areas in use during the day, making the computer less accessible for course participants not working in that room during regular working hours. There were challenges in locating the computers elsewhere due to space and, or security issues. Some (primarily semi-urban) participants used their computer, smartphone, or independent Internet access to bypass this barrier, and many others used the designated computer and internet access outside office hours. Many participants reported that their workload caused challenges in finding time to read materials, complete quizzes, participate in peer or mentored activities, and attend learning sessions. In some facilities, further challenges arose from staff transfers. A few untrained front-line workers and managerial staff were transferred to eDATA-K facilities without being up-to-date on the project or clinical skills. Others were relocated to facilities that had not received a mandate to provide the new services. Those impacted by transfer felt less supported by the leadership.

Another aspect of the learning experience, while not mentioned as challenges per se, could also be improved. Some participants negatively viewed the lack of ethnic diversity or local representation in videos and learning materials. NextGenU.org had included images of diverse ethnicities in the course components it created (introduction, specific learning objectives, instruction related to the open educational resources, etc.). However, the methodology of NextGenU.org is to link to existing high-quality free resources; and most freely available resources for this course were from HICs, representing mostly Caucasian presenters in the videos.

These results and their interpretation were shared and ascertained with county leaders, facility leaders, and course participants through an interactive discussion and presentation at the end of the training.

## Discussion and Conclusion

This study fills a gap in the literature around the impact of using blended-eLearning to teach quality improvement and leadership. While some studies do mention the use of eLearning in quality improvement (Mehta & Sharma, 2018; Suliman et al., 2018), few studies are exploring the impact on trainees’ behaviors and outcomes for patients, something previously cited as needed in research on eLearning (Armstrong et al., 2017; Sinclair et al., 2015) and achieved in this study, with the trainees enabling their facilities to screen 4591 patients of whom 575 received a brief intervention. Those services, not provided in the county before eDATA-K, were sustained in two hospitals and three community clinics in semi-urban and rural areas of an LMIC for the 8-month study period.

The PCQI study confirmed the hypothesis of our theory of change: with adequate support, regardless of the level of education (ranging no formal health education to physicians), location (urban to rural), or type of health facility (hospital versus community clinic), participants can complete the PCQI and affect change in their practices. Participants gained new knowledge, skills, and attitudes necessary to achieve course competencies, integrate and sustain substance-use screening and brief intervention, and affect the care available for those using substances more broadly with the county creating a new SDU specialized clinic offering more than brief interventions, and planning a new inpatient unit. These findings support the acceptability, feasibility, and effectiveness of the PCQI blended-eLearning course and NextGenU.org model in an LMIC context.

In general, the literature supports the viability and advantages of eLearning in health education, including leveraging self-directed learning, providing flexible learning time/modalities that help fit continuous education into busy schedules and increases effectiveness for a variety of learners (Lewis et al., 2014; Sinclair et al., 2015)—attributes all mentioned as valuable to the trainees in this study. This study supports other studies finding that collaborative learning sessions are important for supporting the acquisition and usage of QI (Garcia-Elorrio et al., 2019; Kilo, 1998; Rowe et al., 2018).

Similar to prior findings related to clinical NextGenU.org courses (Clair et al., 2019, 2022b), participants reported a positive experience resulting from a shift in practice patterns, emphasizing mentorship as an important component, as well as the provision of a high-quality course. Further research providing online experiences of “learning sessions” with the addition of remote mentorship to promote scalability would be desirable, as little (though promising) research exists on this topic so far (Mohamed et al., 2014).

The PCQI completion rate (60%) was markedly higher than that of other online courses offered for free (8% on average) (Ho et al., 2015). The participants’ positive experiences with previous NextGenU.org clinical courses contributed to this achievement and the participants’ employer endorsing the training. Other motivational factors are similar to those found previously (Hew & Cheung, 2014; Ho et al., 2015; Song et al., 2004), such as participants’ strong desires for knowledge and skills in this specific topic, obtaining certificates, and receiving a well-designed course. Our previously published findings found that 92% of Kenyan NextGenU.org trainees preferred these blended-eLearning courses to traditional in-person training (Clair et al., 2019). Deficient infrastructure, internet access difficulties, and information technology challenges experienced in this study have been cited in prior studies as impediments to eLearning in both HICs and LMICs (Hew & Cheung, 2014; McKimm et al., 2003; Song et al., 2004; Tarus et al., 2015). However, these issues are addressable, for example, with offline eLearning (Rasmussen et al., 2014), as eDATA-K participants did. Encouragingly, Internet infrastructure, speed, and reliability, as well as access to computers, smartphones, and tablets, continue to improve globally rapidly (Bahia & Suardi, 2019; Dagys et al., 2015; Gomez, 2014; Kebaetse et al., 2014; Marrinan et al., 2015; Parent & Cruickshank, 2009; Sissine et al., 2014; The World Bank, 2020). Accordingly, LMICs are developing more eLearning programs (Atkins et al., 2016; Kebaetse et al., 2014; Marrinan et al., 2015; Suliman et al., 2018; Tarus et al., 2015).

This study’s methodological strengths include using a developmental evaluation approach, with a theory of change guiding course implementation, data collection, and interpretation. The in-depth data collection from multiple sources and methods, including FGDs, KIIs, field observations, and the course eLearning platform data enriched the findings and enabled data triangulation, producing a rich phenomenological explanation of the experience with, and impact of, the PCQI course (Onwuegbuzie et al., 2009; Snape & Spencer, 2003). The iterative process of constant-comparison analysis and double-analysis, plus review by stakeholders and participants, further enhanced our results’ validity, despite response rates on course evaluation of 42% and self-evaluation of 29%.

Despite the small sample size of five facilities with 24 health workers engaged in the course, this study includes diverse health workers (with minimal to extensive prior education levels) in facilities ranging in size and function (from small rural health centers to large peri-urban hospitals), constituting a broad cross-section of Kenyan health system facility types, similar to many other health systems. Although this study occurred in one county, the aforementioned factors support possible transferability to other Kenyan counties, as well as other LMICs as mental health services are tragically globally deficient and can benefit from increased and improved services provided by a range of health workers (Lora et al., 2020; Patel et al., 2013, 2015). Our findings are consistent with prior literature about the topic, further supporting the generalizability of the results. Other studies have supported the effectiveness of eLearning from undergraduate through and continuing medical education (George et al., 2014; Lewis et al., 2014; Liu et al., 2016; Means et al., 2009; Rasmussen et al., 2014), something especially needed for staff in rural and remote areas and those not enrolled in formal programs of study (Sinclair et al., 2015) as was the case with eDATA-K.

Scenarios in which blended eLearning are cost-effective vary, depending on cost for travel, infrastructure (technological versus brick and mortar), course creation, time away from work, number of trainees, etc. (Maloney et al., 2012, 2015; Sandars, 2010; Walsh et al., 2010). The NextGenU.org model assembles open learning resources from universities, governments, professional societies, and peer-reviewed journals to fulfill competencies recommended and published by leading organizations in each field covered by the training. This model minimizes course development cost, while scaling-up the eLearning component cost is low compared to scaling up in-person instruction. For the in-person component, we believe that institutions in LMIC can provide mentors when integrated as part of the regular curriculum or as part of the continuing education system. Individuals in LMIC wanting to take the training on their own have to find a mentor, similarly to students seeking elective placement find academic supervisors. This model is working for the courses developed as part of eDATA-K and other NextGenU courses. The NextGenU.org primary care substance use course has been in high demand internationally after completion of the research program, with another 663 trainees registering from 30 countries, 556 who completed at least module one, and 284 who completed the whole course. The University of Kentucky adopted it for their medical students to continue their education online when university campuses stopped providing in-person learning during COVID-19 pandemic lockdowns. The PCQI course evolved after eDATA-K. NextGenU.org created and taught a full public health leadership course for WHO and included components of the PCQI course in other NextGenU courses related to primary care, public health, mental health, and substance use.

Our study is one of the first assessing blended-eLearning courses’ ability to support the implementation and sustainability of new services in practices in an LMIC and one of the few related to eLearning and QI or leadership (Bonnes et al., 2017; Mehta & Sharma, 2018; Starr et al., 2016; Suliman et al., 2018; Tudor Car et al., 2018).

In conclusion, the results from this study contribute new knowledge on how to train health workers to improve the provision of substance-use care globally. The innovative NextGenU.org blended-eLearning model, as implemented for the PCQI course, effectively provides competency-based education, leading to improvement and expansion of health services, even in resource-constrained settings. The lessons learned and findings from the eDATA-K PCQI study can guide future implementation research, improvement projects, and educational interventions to improve health systems. NextGenU.org continued refining its offering based on the learning from eDATA-K and other experiences in substance use, public health, and primary care, even offering a fully accredited Master of Public Health in collaboration with university partners (Frank, 2020).
